# Acute Effects of Intermittent Versus Continuous Bilateral Ankle Plantar Flexor Static Stretching on Postural Sway and Plantar Pressures: A Randomized Clinical Trial

**DOI:** 10.3390/jcm8010052

**Published:** 2019-01-07

**Authors:** Eva María Martínez-Jiménez, Marta Elena Losa-Iglesias, Jose Ignacio Díaz-Velázquez, Ricardo Becerro-De-Bengoa-Vallejo, Patricia Palomo-López, César Calvo-Lobo, Daniel López-López, David Rodríguez-Sanz

**Affiliations:** 1 Facultad de Enfermería, Fisioterapia y Podología, Universidad Complutense de Madrid, Madrid 28040, Spain; eva.hache2@hotmail.com (E.M.M.-J.); ribebeva@ucm.es (R.B.-D.-B.-V.); david.rodriguez2@universidadeuropea.es (D.R.-S.); 2 Faculty of Health Sciences, Universidad Rey Juan Carlos, Alcorcón 28922, Spain; marta.losa@urjc.es; 3 Departament of Physiotherapy and Podiatry, Premium Madrid, Madrid 28017, Spain; josemarcha@hotmail.com; 4 Department of Nursing, University Center of Plasencia, University of Extremadura, Plasencia 10600, Spain; patibiom@unex.es; 5 Nursing and Physical Therapy Department, Institute of Biomedicine (IBIOMED), Faculty of Health Sciences, Universidad de León, Ponferrada 24401, León, Spain; 6 Research, Health and Podiatry Unit, Department of Health Sciences. Faculty of Nursing and Podiatry, Universidade da Coruña, Ferrol 15403, Spain; daniellopez@udc.es; 7 Faculty of Sport Sciences, Universidad Europea de Madrid, Madrid 28670, Spain

**Keywords:** muscle stretching exercise, postural balance, stabilometry, platform

## Abstract

Background: Postural balance and fall efficacy (self-perceived confidence in performing daily physical activities) have been found to be risk factors associated with falls in older adults. Stretching is one intervention that has been investigated to improve balance and therefore reduce fall risk. Various forms of stretching have been evaluated with different outcomes, but there is a lack of knowledge about the effect of stretching (continuous and intermittent) on plantar pressures and balance. Therefore, the aim of the present study was to analyze the effects of stretching (continuous and intermittent) of the bilateral ankle plantar flexors on plantar pressures and static balance. Methods: A randomized clinical trial was carried out. Forty-eight healthy subjects (42 females and 6 males) were recruited in an outpatient clinic. Subjects were randomly assigned to an intermittent stretching group (five sets of 1 min; 15 s of rest) or a continuous stretching group (2 min of continuous stretching) of the plantar flexors. Plantar pressures and balance using stabilometry were measured before and after stretching. Results: There were significant differences between intermittent and continuous stretching in rearfoot maximum pressure, forefoot surface area, and center of pressure surface area with eyes open. Conclusions: Bilateral intermittent stretching of the ankle plantar flexors was found to be more effective than continuous stretching for the reduction of rearfoot maximum pressure and improved balance.

## 1. Introduction

Worldwide, each year an estimated 646,000 individuals die from falls [1]. Fall efficacy (self-perceived confidence in performing daily physical activities) and postural balance have been identified as risk factors for falls in older adults [2]. Several forms of rehabilitation have previously been used to improve fall risk. Static stretching is one technique of rehabilitation that is commonly used and has been widely researched [3,4,5,6,7,8,9,10,11,12,13,14]. Nevertheless, each form of static stretching may provide different effects and their relationship with balance has not been accurately described.

Two of the more common forms of static stretching include continuous and intermittent modalities. Continuous static stretching has been shown to improve range of motion (ROM) more than intermittent stretching [4]. Specifically, a 2-min continuous stretch increased ROM [5]; however, 5 sets of 1 min stretching with 15 s of rest demonstrated no change in ROM. This type of stretching seems to be duration-dependent, with shorter durations reducing the ROM effects [5].

Static stretching has also been shown to cause a decrease in force production of the muscle. The loss of force with stretching occurs following a minimum of 2 min [5]. When comparing forms of static stretching, it has been found that intermittent stretching (5 sets of 1 min stretching with 15 s of rest) produced greater strength loss compared to continuous stretching (of 5 min) [4]. Both forms of stretching reduce central nervous system pathways conduction, which can reduce force production, but in the case of intermittent stretching there may be other factors that are responsible for prolonged force loss [5].

Postural control is maintained by continuous contraction of anti-gravitational musculature throughout the body. Alterations in the position of the body may lead to variations of load due to the acceleration of gravity and may influence the contraction of the lower back/pelvic (e.g., transversus abdominis) and lower limb flexor muscles. Posture has been deeply analyzed by means of electromyography (EMG), Romberg test analysis, pressure platform analysis, and thermography. The lower limb flexor muscles muscle activation pattern has demonstrated a relationship with static and dynamic balance. Therefore, stretching of the flexor muscles may help to improve the muscle functionality [15,16,17,18,19,20,21].

Currently, there is a lack of consensus on the relationship between lower limb stretching and balance. Several systems may be involved for individuals’ postural control, which may include the neuromuscular, vestibular, visual, and somatosensory systems [6,7]. Behm et al. [9] found a detrimental effect on balance due to an intermittent lower limb stretching protocol. In contrast, Costa et al. [10] found positive effects on dynamic balance due to static stretching. Nevertheless, static and proprioceptive neuromuscular facilitation stretching did not show balance effects, according to Lim et al. [11]. In addition, Chatzopoulos et al. [12] found that static stretching of limbs and arms had negative effects on balance, while dynamic stretching did not show balance effects [13]. Ankle plantar flexors may be considered as postural tonic muscles and can affect postural control by ankle stabilization strategies [14]. Lima et al. [22] also investigated the acute effects of unilateral ankle plantar flexors static- stretching on the center of pressure (COP) during a single-leg balance task, which showed negative balance effects.

Thus, there is a lack of knowledge about the effects of static stretching (continuous and intermittent) on plantar pressures and balance. We hypothesized that static intermittent stretching could improve balance effects. Therefore, the aim of the present study was to analyze the effects of stretching (continuous and intermittent) of the bilateral ankle plantar flexors on plantar pressures and static balance.

## 2. Materials and Methods

### 2.1. Subjects

Forty-eight healthy subjects (42 females and 6 males) were recruited for the study. The demographic data of the study participants were as follows: 32.12 ± 7.60 years old, 166.64 ± 8.13 cm height, and 62.72 ± 8.97 kg weight. All demographic data are shown in Table 1. An ethics committee approved the study, and all subjects gave their written informed consent before participating in this research. Ethical standards in human experimentation contained in the World Medical Association Declaration of Helsinki, the Council of Europe Convention on Human Rights and Biomedicine, the United Nations Educational, Scientific and Cultural Organization (UNESCO) Universal Declaration on the Human Genome and Human Rights, and those of the relevant national bodies and institutions were observed at all times. All subjects were randomly distributed in two different groups: continuous stretching group and intermittent stretching group. Inclusion criteria included: non-trained healthy individuals with no pain [22]. Exclusion criteria included: previous lower extremities surgery; history of lower extremities injury with residual symptoms (pain, “giving-away” sensations) within the last year; evidence of a leg-length discrepancy (difference in distance from the anterior superior iliac spine to the superior surface of the most prominent aspect of the medial malleolus) of more than 1 cm; and evidence of balance deficits (determined by oral questionnaire regarding falls) [22].

### 2.2. Procedures

First, a clinician with ten years of experience performed a baseline balance evaluation to confirm the inclusion and exclusion criteria of each subject using the Balance Evaluation Systems test (BESTest) [23] in order to diagnose equinus foot. All subjects had at least 15 degrees of ankle dorsiflexion [24,25]. For the balance evaluation participants were instructed to remain in a relaxed standing posture with feet shoulder-width apart and positioned at 30° away from the midline on a digital portable pressure sensor platform (Medicapteurs, Balma, France) [26,27,28]. The technical specifications of the pressure platform are shown in the Table 2. Pressure sensor measurements from the platform were accurate to the nearest 0.001 kg/cm^2^. Before each use, auto-calibration was performed.

The protocol started with a pre-stretching evaluation. Participants were then randomly divided into two groups (intermittent stretching group or continuous stretching group) by a random number table provided by the clinician. Each group then performed the prescribed stretching protocol. In order to maintain consistency between groups, the stretching methods were standardized. The subjects climbed on a raised platform and placed the forefoot of both feet on the edge of the platform. They next dropped both heels off the platform to the ground without making contact with the ground [29,30] and held that position to perform a weight-bearing static stretch. The continuous stretching group performed one repetition that was held for two minutes [5]. The intermittent stretching group performed 5 repetitions of 1-min duration stretches with 15 s of rest [4]. During the rest period, the subjects descended from the platform and remained standing. The subjects were asked to quantify a feeling of discomfort during the stretch in both legs. Subjects were instructed to stretch to the point of discomfort [9,16], and this sensation was maintained for all of the stretching [29,31]. The desired point of discomfort (POD) [22] intensity range was 70–90%, considering 0 as “no stretch discomfort at all” and 100% as “the maximum imaginable stretch discomfort”. The POD intensity was recorded during all stretches [22]. Immediately following the completion of the stretching, the testing methods were repeated [22]. All measures were performed at the same hour of the day, between 09:00 and 11:00 h [22]. 

Stabilometry assessment was used and subjects were instructed to stand barefoot on the force platform [26]. The feet were placed at equal distance from the midline [27] and 30 degrees from midline [28]. During all the examinations, the upper limbs were placed in a relaxed position along the body [27]. The subjects were instructed to stand as still as possible for 30 s, with their eyes open (EO), while concentrating on a point at eye level 2-m away [22]. The subjects then repeated the methods with their eyes closed (EC) [26]. Two trials were recorded for each condition [22,26,27] and the order of the conditions was randomized across subjects [26]. Foot plantar pressure and surface area of static footprints were measured during bipodal standing. The foot surface area was then divided into three areas: the rearfoot, midfoot, and forefoot. Two trials were recorded and the average was used for analysis data.

## 3. Measures

### 3.1. Variables

Stabilometry was measured by displacement of the center of pressures in X and Y with eyes open and closed [26], center of pressure (COP) with eyes open and closed, COP area with eyes open and closed, COP antero-posterior (a-p) and medio-lateral (m-lat) directions with eyes open and closed, and COP speed [22].

Static plantar pressure was evaluated by means of maximum pressure, medium pressure and surface area of each aspect of the foot (rearfoot, midfoot, and forefoot).

### 3.2. Statistical Analysis 

All data were explored for normality using the Shapiro Wilks test, and data were considered normally distributed if *p* > 0.05. Descriptive statistical analysis was performed using mean ± SD and a 95% confidence interval. 

The Mann–Whitney U test was performed to examine differences in non-parametric variables. Student’s *t*-test was used for parametric variables. A *p*-value < 0.05 with a confidence interval of 95% was considered statistically significant for all tests (SPSS for Windows, version 20.0; SPSS Inc., Chicago, Illinois, USA).

## 4. Results

All variables were not normally distributed and therefore non-parametric statistics were used (*p* < 0.05), except for socio-demographic characteristics of the sample population. There were no significant differences between groups at baseline (Table 3). There were significant differences between groups after stretching. Specifically, reduced rearfoot maximum pressure, increased forefoot surface area, and reduced surface area of COP with eyes open were shown in the intermittent stretching group compared to the continuous stretching group (Table 4). 

**Figure 1:**
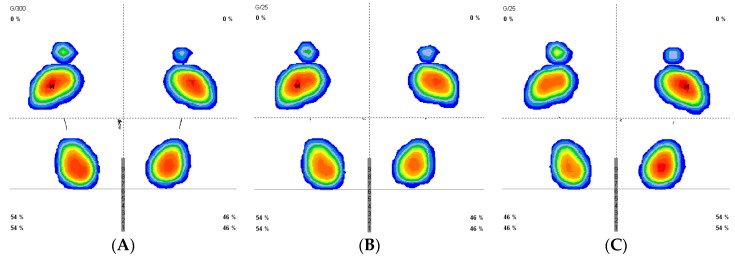
Stabilometry pattern before and after stretching (Previous stabilometry (**A**) post continuous stretch (**B**) and post intermittent stretch(**C**)).

Figure 1 demonstrates the results for the stabilometry before and after continuous and intermittent stretching.

**Figure 2:**
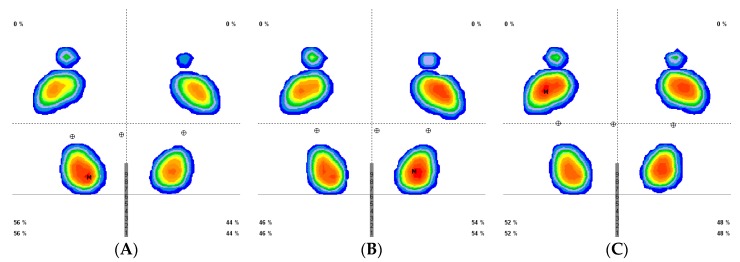
Plantar pressures pattern before and after stretching (prior (**A**), post continuous stretch (**B**), and post intermittent stretch(**C**)).

Figure 2 demonstrates the pattern of the results for plantar pressures prior to stretching and following continuous and intermittent stretching.

## 5. Discussion

This study sought to analyze the effects of stretching (continuous and intermittent) of the bilateral ankle plantar flexors on plantar pressures and static balance.

Both stretching groups (intermittent and continuous) did not demonstrate significant differences during pre-intervention testing. Intermittent stretching demonstrated a reduced maximum pressure in the rearfoot compared to the continuous stretching group. There was also an increase of forefoot surface area in the intermittent group compared to the continuous group. Thus, these results suggest that a greater surface area in the forefoot can reduce the maximum pressure on the rearfoot. 

Ankle equinus generates a large deforming force to the foot and may be considered as a related factor to several foot and ankle conditions, including plantar fasciitis [32], pes planus, hallux abduction valgus, Achilles tendinosis, Charcot’s midfoot collapse, and diabetic ulcerations [33]. Continuous stretching has been used to increase range of movement [4,34,35]. On the other hand, intermittent stretching has been shown to be an effective therapeutic tool for the reduction of muscle stiffness [4,36,37]. 

Stretching exercise leads biomechanical and physiological adaptations related to motor unit excitability improvements, body temperature increases, kinesthetic awareness improvements, and active ROM increases. These biomechanical and neuromuscular modifications may be deeply related to the changes of viscoelastic components of the muscle-tendon units and depressed reflex activity of neural outputs. This phenomenon is called viscoelastic stress relaxation and demonstrates that the muscle–tendon unit is affected during stretching activity [3]. 

Continuous stretching effects may be also related to a muscle compliance increase, which increases the time and decreases the force for myofibril-unit contraction during muscular elements activation. Continuous stretching may affect the maximal force production and reduce muscle stiffness and muscle activation, as well as generating an acute increase of ROM. These modifications have been related to an increased tolerance to the imposed stretching activity [9,29]. Nevertheless, intermittent stretching may enhance muscular condition by preserving muscle-tendon unit stiffness and may improve the recovery of the vascular, nervous, muscle–tendon unit, and metabolic systems, as well as stretching tolerance.

Both continuous and intermittent stretching interventions have been related to dynamic balance and postural control. Balance and joint position sense are proprioceptive parameters that depend on contributions from visual, vestibular and peripheral receptors that are found in skin, joints, muscles and ligaments [12]. 

Proprioception may provide conscious and subconscious responses for body posture and motion, being essential for lower limb joints functioning in order to maintain optimal balance control during daily physical activities. Joint position sense may be considered as an aspect of proprioception that plays an important role in functional dynamic stability of joints through the action of the muscles and ligaments around them, which seems to be influenced by ROM modifications [10].

Therefore, intermittent stretching use has been recommended for pathologies that present an increased heel pressure such as heel pain (Sever’s disease [20,21], fasciitis [32]) as well as those with increased pressure in the forefoot (fasciitis [32], diabetic foot ulcers [33,38], metatarsalgia [39], Achilles tendinopathy [40]). Gajdosik et al. [34] found an increased ROM after ten static wall intermittent stretches held for 15 s in each repetition, five times per week for 6 weeks. Nevertheless, more studies are necessary to examine the effect of intermittent stretching on equine feet and find the most appropriate protocol to reduce plantar pressures. 

Morrin and Redding in 2013 [41] found that continuous static stretching on the hamstrings in dancers did not show detrimental effects on balance. A study by Behm et al. in 2004 [9] found significant decreases in static balance scores following an intermittent stretching protocol (5-min cycle warm-up, three stretches to the point of discomfort, 45 s each with 15-s rest periods for each muscle group of the lower limb). Depending on the muscle studied, different acute effects of static stretching may occur [42]. It is important to consider that the results obtained in calf muscles may not be generalized to other muscle groups. 

This fact may explain the results of our study, which found intermittent stretching reduced COP surface area with the eyes open, while changes were not observed after continuous stretching. 

Most authors suggest that changes in balance after stretching might be related to changes in both proprioception and mechanical outputs (influencing the musculotendinous unit stiffness and affecting the ability to adapt adequately to the stability challenges), [9,10,12,41]. The findings for COP surface area with the eyes open following intermittent stretching may be related to an increase in surface area of the forefoot following stretching, leading to an increase in proprioceptive stimulus from the skin receptors. This can also suggest that intermittent stretching may reduce force and inhibit central nervous system pathways conduction compared to continuous stretching [42] due to the fact that the modifications in osteotendinous and Golgi reflex in plantar flexors have been found to produce less static balance influence than other muscles, such as the tibialis anterior which has been highly related to center of pressure movement compared to the plantar flexor muscles in bipodal standing [43].

## 6. Conclusions

The force platform assessment demonstrated that bilateral intermittent stretching of the ankle plantar flexor muscles seems to be more effective than continuous stretching for the reduction of rearfoot maximum pressure and balance improvement.

## Figures and Tables

**Table 1:** Socio-demographic characteristics of the sample population.

Variable Total (*n* = 24)	Total Group Mean ± SD (CI 95%)	Continuous Group Mean ± SD (CI 95%)	Intermittent Group Mean ± SD (CI 95%)	*p*-Value *
Age (years)	32.12 ± 7.60 (29.08–35.16)	32.20 ± 8.08 (28.97–35.44)	32.04 ± 7.28 (29.18–34.89)	0.940
Weight (kg)	62.72 ± 8.97 (59.14–66.31)	62.77 ± 9.52 (58.96–66.57)	62.68 ± 8.58 (59.32–66.05)	0.975
Height (cm)	166.64 ± 8.13 (163.39–169.90)	166.20 ± 8.43 (162.83–169.58)	167.08 ± 7.98 (163.95–170.21)	0.714
BMI (kg/m^2^)	22.58 ± 2.75 (21.48–23.69)	22.71 ± 2.90 (21.55–23.87)	22.46 ± 2.66 (21.41–23.50)	0.753
Size of shoe	38.87 ± 2.32 (37.94–39.80)	38.81 ± 2.26 (37.90–39.72)	38.93 ± 2.43 (37.98–39.89)	0.855

Abbreviations: kg, kilograms; cm, centimeters; BMI, body mass index; SD, standard deviation; CI 95%, confidence interval 95%. In all the analyses, *p* < 0.05 (with a 95% confidence interval) was considered statistically significant. *p*-values are from Student’s *t*-test *.

**Table 2:** Technical specifications of the pressure platform.

Specification	Description
Size (length × width × height)	530 × 600 × 45 mm
Thickness	4 mm
Active surface	400 × 400 mm
Weight	6.8 kg
Sensors	Calibrated resistive
Sensor	8 × 8 mm
Sensor thickness	0.15 mm
No. of sensors	2304 (48 × 48)
Permissible temperature	−40 °C to 85 °C
Sensor pressure (minimum/maximum)	0.4 N/m^2^ (0.0004 kPa)/100 N/m^2^ (0.1 kPa)
Type of PC interface/platform	Universal Serial Bus (USB)
Supply	USB cable
Data acquisition frequency	200 images/s
Vertical force recording	60 Hz
Operating system required	Windows XP, Vista, or 7

**Table 3:** Stabilometry and static footprints variables before bilateral intermittent and continuous stretching.

Variable	Intermittent Group Pretest Values (*n* = 20) Mean ± SD (CI 95%)	Continuous Group Pretest Values (*n* = 20) Mean ± SD (CI 95%)	*p*-value *
Rearfoot maximum pressure (kPa)	106.24 ± 21.36 (97.00–115.48)	105.52 ± 24.08 (95.02–105.93)	0.918
Rearfoot medium pressure (kPa)	39.61 ± 6.51 (36.79–42.43)	41.59 ± 8.96 (37.71–45.46)	0.483
Rearfoot surface (cm^2^)	85.84 ± 10.51 (81.30–90.39)	84.71 ± 10.33 (80.25–89.18)	0.828
Midfoot maximum pressure (kPa)	13.05 ± 14.82 (6.64–19.46)	11.18 ± 11.69 (6.12–16.23)	0.865
Midfoot medium pressure (kPa)	5.72 ± 6.10 (3.08–8.36)	5.63 ± 5.68 (3.17–8.09)	0.966
Midfoot surface (cm^2^)	16.71 ± 19.08 (8.46–24.96)	17.93 ± 20.07 (9.25–26.61)	0.787
Forefoot maximum pressure (kPa)	69.41 ± 13.19 (63.71–75.12)	70.16 ± 10.36 (65.68–74.64)	0.606
Forefoot medium pressure (kPa)	25.54 ± 6.07 (22.91–28.16)	25.23 ± 2.77 (24.03–26.43)	0.338
Forefoot surface(cm^2^)	94.15 ± 17.75 (86.47–101.82)	90.02 ± 12.02 (84.81–95.22)	0.496
X displacement eyes open (mm)	6.91 ± 6.14 (4.26–9.57)	8.34 ± 9.01 (6.49–10.19)	0.083
Y displacement eyes open (mm)	16.68 ± 9.89 (12.40–20.95)	19.32 ± 9.01 (15.43–23.22)	0.359
Surface Eyes Open (mm^2^)	13.35 ± 9.58 (9.21–17.50)	9.83 ± 7.12 (6.75–12.92)	0.180
Medium speed of the laterolateral displacement. Eyes open (mm/s)	1.20 ± 0.27 (1.08–1.32)	1.16 ± 0.28 (1.03–1.28)	0.657
Medium speed of the anteroposterior displacement. Eyes open (mm/s)	0.98 ± 0.26 (0.87–1.10)	1.04 ± 0.39 (0.87–1.21)	0.542
X displacement eyes closed (mm)	7.74 ± 5.05 (5.55–9.92)	7.61 ± 4.40 (5.71–9.52)	0.926
Y displacement eyes closed (mm)	17.08 ± 9.93 (12.78–21.37)	21.34 ± 9.46 (17.24–25.43)	0.164
Surface eyes closed (mm^2^)	25.96 ± 14.77 (19.57–32.34)	32.44 ± 52.54 (9.71–55.16)	0.599
Medium speed of the laterolateral displacement. Eyes closed (mm/s)	1.39 ± 0.36 (1.23–1.55)	1.37 ± 0.42 (1.19–1.56)	0.788
Medium speed of the anteroposterior displacement. Eyes closed (mm/s)	1.48 ± 0.58 (1.22–1.73)	1.41 ± 0.70 (1.10–1.72)	0.332

Abbreviation: kg (kilograms); cm (centimeters), cm^2^ (centimeters^2^), SD (standard deviation), CI 95% (confidence interval 95%). In all the analyses, *p* < 0.05 (with a 95% confidence interval) was considered statistically significant. *p*-values are from Mann–Whitney U test *.

**Table 4:** Stabilometry and plantar pressure variables after bilateral intermittent and continuous stretching.

Variable	Intermittent Group Posttest Values (*n* = 20) Mean ± SD (CI 95%)	Continuous Group Posttest Values (*n* = 20) Mean ± SD (CI 95%)	*p*-Value *
Rearfoot maximum pressure (kPa)	87.56 ± 22.77 (77.71–97.41)	99.39 ± 18.76 (91.28–107.51)	0.019
Rearfoot medium pressure (kPa)	33.84 ± 7.44 (30.62–37.06)	37.19 ± 5.38 (34.86–39.51)	0.105
Rearfoot surface (cm^2^)	81.86 ± 12.11 (76.62–87.10)	83.54 ± 13.11 (77.87–89.21)	0.703
Midfoot maximum pressure (kPa)	14.73 ± 13.98 (8.68–20.78)	15.58 ± 14.85 (9.16–22.01)	0.983
Midfoot medium pressure (kPa)	7.05 ± 6.55 (4.21–9.88)	7.54 ± 6.22 (4.85–10.23)	0.761
Midfoot surface (cm^2^)	20.78 ± 18.67 (12.70–28.85)	20.56 ± 19.08 (12.31–28.81)	0.957
Forefoot maximum pressure (kPa)	74.16 ± 15.62 (67.41–80.92)	73.14 ± 15.38 (66.48–79.79)	0.332
Forefoot medium pressure (kPa)	26.98 ± 4.29 (25.12–28.84)	26.72 ± 6.73 (23.81–29.63)	0.322
Forefoot surface (cm^2^)	105.23 ± 18.32) (97.31–113.16)	97.36 ± 12.98 (91.75–102.98)	0.038
X displacement eyes open (mm)	8.48 ± 5.23 (6.22–10.75)	7.32 ± 4.89 (5.20–9.44)	0.353
Y displacement eyes open (mm)	15.89 ± 8.39 (12.26–19.51)	18.29 ± 10.84 (13.60–22.98)	0.409
Surface eyes open (mm^2^)	6.34 ± 4.08 (4.57–8.10)	11.02 ± 8.58 (7.31–14.74)	0.031
Medium speed of the laterolateral displacement. Eyes open (mm/s)	1.19 ± 0.31 (1.05–1.32)	1.35 ± 0.71 (1.04–1.66)	0.910
Medium speed of the anteroposterior displacement. Eyes open (mm/s)	1.15 ± 0.65 (0.87–1.43)	1.02 ± 0.57 (0.77–1.27)	0.474
X displacement eyes closed (mm)	7.97 ± 5.86 (5.43–10.50)	6.51 ± 4.09 (4.74–8.29)	0.557
Y displacement eyes closed (mm)	15.54 ± 9.53 (11.42–19.66)	16.90 ± 9.62 (12.74–21.06)	0.640
Surface eyes closed (mm^2^)	18.24 ± 12.29 (12.93–23.56)	23.17 ± 16.36 (16.09–30.24)	0.167
Medium speed of the laterolateral displacement. Eyes closed (mm/s)	1.54 ± 0.47 (1.34–1.75)	1.43 ± 0.85 (1.07–1.80)	0.051
Medium speed of the anteroposterior displacement. Eyes closed (mm/s)	1.79 ± 0.87 (1.42–2.17)	1.89 ± 2.31 (0.89–2.89)	0.083

Abbreviation: kg (kilograms); cm (centimeters), cm^2^ (centimeters^2^); SD (standard deviation), CI 95% (confidence interval 95%). In all the analyses, *p* < 0.05 (with a 95% confidence interval) was considered statistically significant. *p*-values are from Mann–Whitney U test *.
